# Pharmacogenetics of the Central Nervous System—Toxicity and Relapse Affecting the CNS in Pediatric Acute Lymphoblastic Leukemia

**DOI:** 10.3390/cancers13102333

**Published:** 2021-05-12

**Authors:** Judit C. Sági, András Gézsi, Bálint Egyed, Zsuzsanna Jakab, Noémi Benedek, Andishe Attarbaschi, Stefan Köhrer, Jakub Sipek, Lucie Winkowska, Marketa Zaliova, Stavroula Anastasopoulou, Benjamin Ole Wolthers, Susanna Ranta, Csaba Szalai, Gábor T. Kovács, Ágnes F. Semsei, Dániel J. Erdélyi

**Affiliations:** 1 Department of Genetics, Cell and Immunobiology, Semmelweis University, H-1089 Budapest, Hungary; sagi.judit@med.semmelweis-univ.hu (J.C.S.); szalai.csaba@med.semmelweis-univ.hu (C.S.); 2 Department of Measurement and Information Systems, Budapest University of Technology and Economics, H-1111 Budapest, Hungary; gezsi.andras@gmail.com; 3 MTA-SE Immune-Proteogenomics Extracellular Vesicle Research Group, Semmelweis University, H-1089 Budapest, Hungary; 4 2nd Department of Pediatrics, Semmelweis University, H-1094 Budapest, Hungary; egyed.balint@med.semmelweis-univ.hu (B.E.); jakab.zsuzsanna@med.semmelweis-univ.hu (Z.J.); kovacs.gabor1@med.semmelweis-univ.hu (G.T.K.); 5 Department of Pediatrics, Pécs University, H-7623 Pécs, Hungary; noemi.benedek@gmail.com; 6 Department of Pediatric Hematology and Oncology, St. Anna Children’s Hospital, Medical University of Vienna, A-1090 Vienna, Austria; andishe.attarbaschi@stanna.at; 7 Renate Panzer-Grümayer: Leukemia Biology Group, Children’s Cancer Research Institute, A-1090 Vienna, Austria; stefan.koehrer@ccri.at; 8 Department of Paediatric Haematology and Oncology, 2nd Faculty of Medicine, Charles University and University Hospital Motol, CZ-150 06 Prague, Czech Republic; jakub.sipek@fnmotol.cz (J.S.); lucie.winkowska@lfmotol.cuni.cz (L.W.); marketa.zaliova@lfmotol.cuni.cz (M.Z.); 9 CLIP—Childhood Leukemia Investigation Prague, Department of Paediatric Haematology and Oncology, 2nd Faculty of Medicine, Charles University and University Hospital Motol, CZ-150 06 Prague, Czech Republic; 10 Childhood Cancer Research Unit, Department of Women’s and Children’s Health, Karolinska Institutet, S-17177 Stockholm, Sweden; stavroula.anastasopoulou@ki.se (S.A.); susanna.ranta@ki.se (S.R.); 11 Astrid Lindgren Children’s Hospital, Karolinska University Hospital, S-14186 Stockholm, Sweden; 12 Department of Paediatrics and Adolescent Medicine, Rigshospitalet, DK-2100 Copenhagen, Denmark; benjamin.ole.wolthers@regionh.dk; 13 Central Laboratory, Heim Pál Children’s Hospital, H-1089 Budapest, Hungary

**Keywords:** encephalopathy, CNS toxicity, CNS relapse, PRES, genetic polymorphisms, childhood leukemia

## Abstract

**Simple Summary:**

Despite recent improvements in cure rates, pediatric acute lymphoblastic leukemia (ALL) patients remain at risk to develop relapse disease or suffer from therapy-associated side effects. Over 5% of adverse events appear in the central nervous system (CNS) and can impact survival or quality of life of the patients. Inherited genetic variations are possible predictive factors for these adverse events. This retrospective study aimed to investigate if inherited genetic variations in genes encoding drug-metabolizing enzymes and drug transporters localized in the blood-brain barrier are predictive for CNS events. Our results suggest that certain *ABCB1*, *ABCG2* and *GSTP1* gene polymorphisms influence CNS toxicity and CNS relapse. A more effective drug-clearance could lead to less toxicity but contribute to a higher chance of relapse and vice versa. Genetic variants in *ABCB1*, *ABCG2* or *GSTP1* genes are promising candidates for personalized medicine.

**Abstract:**

Despite improving cure rates in childhood acute lymphoblastic leukemia (ALL), therapeutic side effects and relapse are ongoing challenges. These can also affect the central nervous system (CNS). Our aim was to identify germline gene polymorphisms that influence the risk of CNS events. Sixty single nucleotide polymorphisms (SNPs) in 20 genes were genotyped in a Hungarian non-matched ALL cohort of 36 cases with chemotherapy related acute toxic encephalopathy (ATE) and 544 controls. Five significant SNPs were further analyzed in an extended Austrian-Czech-NOPHO cohort (*n* = 107 cases, *n* = 211 controls) but none of the associations could be validated. Overall populations including all nations’ matched cohorts for ATE (*n* = 426) with seizure subgroup (*n* = 133) and posterior reversible encephalopathy syndrome (PRES, *n* = 251) were analyzed, as well. We found that patients with *ABCB1* rs1045642, rs1128503 or rs2032582 TT genotypes were more prone to have seizures but those with rs1045642 TT developed PRES less frequently. The same SNPs were also examined in relation to ALL relapse on a case-control matched cohort of 320 patients from all groups. Those with rs1128503 CC or rs2032582 GG genotypes showed higher incidence of CNS relapse. Our results suggest that blood-brain-barrier drug transporter gene-polymorphisms might have an inverse association with seizures and CNS relapse.

## 1. Introduction

Cancer is the leading cause of illness-linked deaths in childhood in the developed countries [1]. The most common pediatric malignancy is acute lymphoblastic leukemia (ALL). Its effective chemotherapy regimens yield more than 90% long term survival rates in developed countries [2]. Although, adverse drug reactions are still a challenge [3]. Pharmacokinetics and pharmacodynamics of the drugs are also influenced by germline gene variants, some already indicated in drug labels [4,5]. The most important genetic risk factors are already described in pediatric ALL as well, however, they failed to reliably predict prognosis [6]. Relapse, and toxicity-related deaths still limit outcome, therefore understanding their genetic background and finding predictive biomarkers are still important [7,8]. Approximately 5–15% of children with ALL were reported to suffer from acute central nervous system (CNS) toxicity during the treatment, while isolated and/or combined CNS relapse occurred in 3–8% [9,10,11]. Neurotoxicity is the second most common adverse event to trigger dose-reduction of chemotherapeutic agents [12]. However, there are limited number of publications investigating prognostic factors of acute CNS toxicity or pharmacogenetics of CNS relapse in ALL [13,14,15].

There are a few suggested pharmacogenetic risk factors for relapse or neurotoxicity in ALL. SNPs in a MTX (methotrexate) pathway enzyme, methylene tetrahydrofolate reductase *(MTHFR*) were reported to associate with neurotoxicity [16], and so were CYP3A5*1/*3 alleles (cytochrome P450 family 3 subfamily A member 5) that play a role in vincristine metabolism [17]. Central nervous system toxicity during cancer therapy was also associated with genetic variants of *ABCB1*, *ABCG2* and *GSTP1* (glutathione S-transferase 1) [18,19,20]. Variants in *GSTP1* and *ABCB1* genes were also associated with CNS relapse or overall relapse [15,21]. Vincristine-pathway genes were not associated with relapse of ALL, but with peripheral neurotoxicity [22].

Symptoms of CNS toxicities vary based on etiology and the affected areas. Adverse complications within the brain may cause acute, subacute, or delayed encephalopathy [6]. Toxic encephalopathy may be reversible or permanent and can lead to neurocognitive impairment [23]. The diagnosis of acute toxic encephalopathy is based on clinical features and specific MRI findings [24]. Differential diagnosis requires the exclusion of peripheral neuropathy, CNS infection, intracranial vascular events, CNS malignancy, effect of sedative medications, or metabolic disturbances e.g., liver failure [25]. A nomenclature established in 2016 defined some typical CNS toxic events of childhood ALL therapy: methotrexate-related stroke-like syndrome (SLS), seizures, posterior reversible encephalopathy syndrome (PRES), and depressed level of consciousness [26]. PRES and SLS have specific clinical and/or radiological patterns. Suspicion for PRES or SLS might be triggered by any CNS symptom with unique MRI lesions [10,26]. Chemotherapy related neurotoxicity in children with ALL appeared most often among females and at younger age [27]. Moreover, it was also described that risk for PRES and seizures is higher in older children (>10 years) [28,29]. Toxicity of intrathecal chemotherapy was associated with age above 3 years in a different study [30]. However CNS involvement did not associate with MTX neurotoxicity [31].

Patients with relapsed ALL face unfavorable outcome, their 5-year overall or event-free survival (OS, EFS) varies around 30–50% [32,33]. Approximately 30% of patients with relapsed ALL have CNS leukemia (combined or isolated) [15,34]. Repeated doses of intrathecal chemotherapy (CNS treatment of CNS negative ALL patients) [27,34] in combination with CNS directed systemic chemotherapy has reduced the CNS relapse rate to 5% for the nineties [35]. Intrathecal dose intensification by CNS status at diagnosis could improve the prevention of CNS relapses [36,37,38,39,40,41].

Systemic and CNS directed treatment of ALL are known to be neurotoxic both in the short and in the long term [27,34,42]. Vincristine, methotrexate, cytarabine, l-asparaginase, iphosphamide, and glucocorticoids (prednisone and dexamethasone) are thought to exert the most acute adverse effects in the CNS [13,27]. It is usually hard to find single cause-effect relationships as multi-agent chemotherapy cycles are used, and other factors like drug-drug interactions, cranial irradiation, CNS-infiltration must also be considered [13]. Therefore, biomarkers for predicting CNS complications are much needed [34].

In 2007, we published a study on BBB pharmacogenetics of CNS toxicity in childhood ALL [20]. Acute toxic encephalopathy (ATE, any ≥ grade 3 CNS toxicity directly evoked by chemotherapy) was found to be more frequent among patients homozygous for the *ABCB1* rs1045642 T allele; and the association was stronger with a combination of *ABCB1* rs1045642 TT and *ABCG2* rs2231142 CA/AA genotypes. In this study, our aims were to (1) reexamine this question on a larger patient cohort, with an extended set of SNPs relevant in pharmacogenetics; and (2) to examine the association of the same SNPs with leukemia CNS relapse. We hypothesized that a functional SNP leading to a higher concentration of chemotherapeutics in the brain would increase the risk of CNS toxicity but reduce the chance of CNS relapse, or vice versa.

## 2. Materials and Methods

### 2.1. Patients

We enrolled to all study cohorts children treated for frontline ALL, at ages 0–18 years (1–18 years for toxicity analyses to avoid infant patients on different chemotherapy regimens; 0–18 years for analyzing relapses) at diagnosis in Hungary, Austria, Czech Republic and in the NOPHO group (Denmark, Norway, Sweden, Finland, Iceland, Lithuania, Estonia) [43]. We excluded children with any previous chemotherapy, any major deviations from ALL protocol to focus on pharmacogenetic effects. Clinical data were collected from the medical records of the patients retrospectively. Data collection sheets of the PdL ‘Retrospective Investigation of Children with ALL/LBL with Central Neurotoxicity Related to Therapy’ study were used (with complements to Christina Halsey and the Ponte di Legno Toxicity Working Group) as all four contributing groups are participating in that ongoing study. See Table 1, Table 2 and Table 3, and Table S7 for characteristics of cohorts.

The two main studied phenotypes were adverse CNS symptoms called acute encephalopathy (AE) and CNS relapse. The definition of AE was any evolving adverse CNS symptom at least grade 3 as per Common Terminology Criteria for Adverse Events (CTCAE) v.4.0 occurring after the first dose of anti-leukemic treatment but within 3 weeks after the last dose of i.v. chemotherapy [44]. Patients with preceding CNS diseases; with uncertain, or mild neurologic symptoms were excluded from all analyses targeting neurotoxicity.

**Figure 1:**
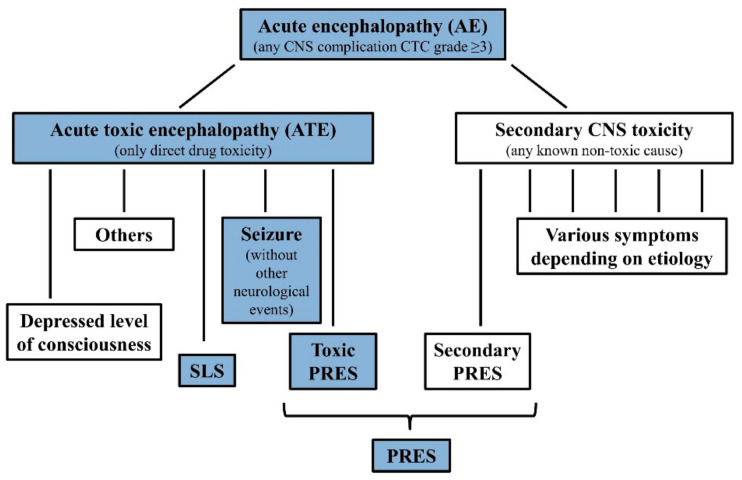
Classification of CNS toxicities during childhood ALL therapy.

CNS adverse events with no known secondary etiology are defined as acute toxic encephalopathy (ATE.), as subgroup of AE. AE cases with identified underlying systemic causes including cerebrovascular events, CNS infections, actual CNS leukemia not in remission, metabolic alterations (e.g., severe electrolyte disturbance, hepatic encephalopathy, hypoglycemia or diabetic ketoacidosis) or insufficient CNS circulation (e.g., hypertensive encephalopathy, increased intracranial pressure, severe anemia or sepsis with hypotension or hypoxia) possibly causing CNS symptoms were excluded. See more details in Supplementary Materials Patient Criteria. Hence, only events with suspected direct chemotherapy-related CNS adverse toxic effects were stratified as drug-induced ATE. These patients could be classified into the overlapping Delphi consensus definitions of stroke-like syndrome (SLS), seizures without other neurological events, depressed level of consciousness, posterior reversible encephalopathy syndrome (PRES), however, these symptoms could also be observed with known secondary cases in the AE cohort [26] (Figure 1). Two controls per case were enrolled. Controls were pediatric patients with ALL who experienced none of these events, had no comorbidities, medical history, or co-medication that may have influenced the occurrence of CNS complications or drug pharmacokinetics.

We categorized each event of AE according to four different types of chemotherapy cycles taking into account during or after what type of chemotherapy the CNS complication evolved (see more details in Table S1b).

Boxes of studied phenotypes are highlighted with blue background. Note: symptoms of ATE subgroups may overlap, see definitions at Reference [26]. Further rare manifestations of ATE are not demonstrated in the Figure 1, e.g., ataxia, extrapyramidal movements, steroid evoked psychosis, etc. Secondary CNS toxicities may present with different, similar or same symptoms as ATE. E.g., PRES can be caused by hyponatremia or by severe hypertension, but may also present without these.

For the CNS relapse case-control analysis, 1st ALL relapse cases were selected, both isolated CNS and combined medullary plus CNS, and other extramedullary plus CNS relapses. Three controls per one case were matched: two non-relapsed patients with ALL and one isolated BM first relapse case. See Supplementary Materials Patient Criteria for details.

### 2.2. Study Design, Overview

Following the 2007 publication, further Hungarian ALL patients were enrolled between 2005 and 2015. Sixty SNPs in 20 genes encoding drug-metabolizing enzymes and transporters were studied on the whole 1990–2015 Hungarian non-matched patient cohort (*n* = 580). To validate prior results, we organized a European case-control matched cohort with Austrian, Czech, and Nordic Society of Pediatric Hematology and Oncology (NOPHO) groups for validation of the ATE—genotype associations found in the Hungarian population (validation cohort: 107 ATE cases and 211 controls). SLS, seizure without other neurological events, toxic PRES, altered consciousness, and their overlap cases were requested, and two matched controls for each case. The same enrolment criteria were used for all of the study groups when selecting patients for the Joined validation cohort. In the same study, we also examined another AE phenotype, PRES, which included cases with toxic or secondary causes (82 PRES cases, 169 controls). Together, the four groups had enough cases to test for the effect of the same SNPs on CNS relapse, as well (86 CNS relapse cases (isolated or combined), 105 isolated bone-marrow (BM) relapse cases, 129 controls). The number of patients to be involved was designed based on the results of the discovery population with the statistical power of 0.8. For the demonstration of the study elements of CNS toxicity in a flow chart see Figure 1.

**Figure 2:**
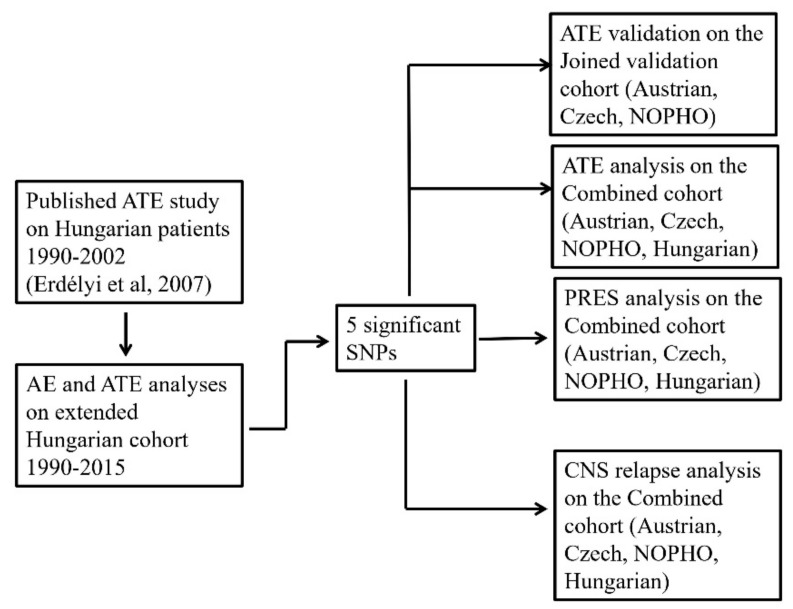
Study design.

Throughout the paper, by ‘Joined cohort’, we mean the Austrian-Czech-NOPHO case-control ATE validation population. By ‘Combined cohort’ of ATE or PRES or CNS relapse, we mean the matched study populations of all the four study groups (Hungarian, Austrian, Czech and NOPHO), respectively. See Figure 2.

### 2.3. Ethical Considerations

The study was conducted according to the principles expressed in the Declaration of Helsinki for all nations. Written informed consent was requested from all patients or the parents or guardians of the minors involved in the study. The study was approved by the ethical committees in the participating countries. These are: Ethics Committee of the Medical University of Vienna on 3 August 2010 (No. 641/2010) (Austrian patients); Ethics Committee of University Hospital Motol (approval file number NV15-30626A, approved in August 2014) (Czech patients); Ethics Committee of the Hungarian Medical Research Council (approval file number 12988-52-1018/-EKU, Date: 29 September 2003, 23310–1/2011/EKU, Date: 19 January 2012, ad. 60106-1/2015/EKU, Date: 21 December 2015) (Hungarian patients). The database containing phenotype data was approved by the Swedish Ethical Review Authority (731-10 (date 17 January 2011), the regional ethical review board of The Capital Region of Denmark (H-2-2010-022), the Danish Data Protection Authorities (j.nr.: 2012-58-0004), and by relevant regulatory authorities in all participating countries. Genotype data were stored at the Technical University of Denmark’s server Computerome (NOPHO patients).

### 2.4. Laboratory Methods

DNA was isolated from peripheral blood taken in remission and 60 SNPs (Table S1a) of drug-metabolizing or transporting genes were selected and genotyped in the Hungarian population. The main features of the studied SNPs are summarized in Table S1a. Genotype data of 5 SNPs were requested from collaboration partners. In the Austrian cohort genotyping was performed via Sanger sequencing of remission bone marrow samples (see Table S1c for primer sequences). Major proportion of Czech patients was genotyped using KASPar (KBioscience Competitive Allele-Specific Polymerase chain reaction)-on-Demand prevalidated assays (LGC Biosearch Technologies, Hoddesdon, United Kingdom). Minor proportion was genotyped using the single nucleotide polymorphism arrays (HumanOmni Express BeadChip from Illumina, San Diego, CA, United states and CytoScan HD arrays from Affymetrix, Santa Clara, CA, United States) as described previously [45]. Genotyping of Hungarian patients was conducted using TaqMan^®^ OpenArray™ Genotyping System (Thermo Fisher Scientific, Waltham, MA, United States) or using KASPar-on-Demand prevalidated assays (LGC Biosearch Technologies, Hoddesdon, United Kingdom) following the manufacturer’s instructions as described earlier [46]. The genotyping of NOPHO patients were performed using Omni2.5exome-8-BeadChip arrays (Illumina, San Diego, CA, United States) and described in detail in the article of Wolthers et al. [47] and Hojfeldt et al. [48].

### 2.5. Statistical Analysis

In the Hungarian cohort, multivariate logistic regression models (for case-control analysis) and Cox proportional hazards regression models (for survival analysis) were used to investigate the influence of genetic polymorphisms on the neurological symptoms affecting the brain and CNS relapse. In the Joined cohort and during the analysis of the Combined cohort, conditional logistic regression models (for case-control analysis on cohorts with matched controls) and Cox proportional hazard regression models for nested case-control data (for survival analysis on cohorts with matched controls) were used. We calculated the OS, EFS in every disease cohort (AE sub-phenotypes, CNS relapse). Fisher exact test was used to evaluate the association between NOPHO or BFM-protocols and occurrence of the studied phenotypes. Detailed study constructions of CNS events are shown in Figure 2. The number of cases with depressed level of consciousness was below 10, so we did not analyze this sub-phenotype separately. Confounders used in analyses are shown in Table S1b. Allele frequencies were estimated by allele counting and tested for deviation from Hardy-Weinberg equilibrium (HWE) by the on-line software (Tests for deviation from Hardy-Weinberg equilibrium *p*. https://ihg.gsf.de/cgi-bin/hw/hwa1.pl (accessed on 27 March 2021)). Significant violation of HWE was considered if *p* ≤ 1.13 × 10^−2^. Confidence intervals (CI) or hazard ratios (HR) were calculated at the 95% level. The analyses were performed studying the genotypes separately (11 vs. 12; 11 vs. 22), using dominant (11 vs. 12/22) or recessive (11/12 vs. 22) models, with the common homozygotes signed as 11 and the rare (2) allele supposed to be dominant. If the number of rare homozygote patients was *n* ≤ 10, we merged them with heterozygote patients for the analyses. Genotype combinations were determined based on the results of these merged groups. Multiple testing corrections were performed using the Benjamini-Hochberg false discovery rate (FDR) method with a type I error rate of 13% [49,50]. Alpha levels of *p* ≤ 1.13 × 10^−2^ were considered significant after FDR correction in multiple testing for the studied SNPs (with 465 analyses performed for 60 or 5 SNPs and each phenotype). Results reported without mentioning the used model were studied in additive model. Analyses were performed using IBM SPSS Statistics 25.0 (IBM Corporation, Armonk, NY, United States) and R statistical software (version 3.6.3, R Foundation for Statistical Computing, Vienna, Austria). Conditional logistic regression analyses were performed by the clogit function of the survival package of R [51]. Cox proportional hazards regression analyses for nested case-control data were performed by the multipleNCC package [52]. Power analysis was conducted by PS: Power and Sample Size Calculation 3.1.2.

## 3. Results

### 3.1. Chemotherapy Related Adverse Neurological Symptoms

#### 3.1.1. Case-Control Analyses

Acute encephalopathy and its sub-phenotype, ATE, were first studied in the Hungarian discovery cohort. The following genotypes below were found to associate with both AE and ATE: *ABCB1* rs1045642 TT, rs1128503 TT and the combination of *ABCB1* rs1045642 TT genotype with *ABCG2* rs2231142 CA or AA genotypes. *GSTP1* rs1695 AG + GG genotype associated with decreased risk for AE and ATE in this cohort. *ABCB1* rs2032582 TT associated with ATE only. The other examined 55 SNPs showed no significant association with ATE. The summary of the results is shown in Tables S2a and S4a. When analyzing the 5 selected SNPs and ATE in the Joined validation cohort (Austrian, Czech and NOPHO case-control cohort), none of the associations could be confirmed. The relation of *GSTP1* rs1695 and ATE was actually the opposite of that found in the Hungarian cohort, while tests with the *ABC* SNPs were largely non-significant (see Tables S2a and S4b).

The Combined cohort of patients including both the matched Hungarian ATE cohort and the Joined validation cohort was large enough for more detailed analyses of neurotoxicity phenotypes: seizure without other neurological events, SLS, and toxic PRES. T alleles of *ABCB1* rs1045642, rs1128503 and rs2032582 polymorphisms appear to be associated with seizures, and particularly with seizures during Induction-like chemotherapy cycles (see Tables S2a and S4c). On the other hand, the *ABCB1* rs1045642 CT genotype might be protective against PRES and toxic PRES. In addition to the genetic variations, CNS 2 status was also predictive for PRES (OR = 5.08, CI 95% (2.10–12.29)) (see Tables S2a and S4c,d). PRES and toxic PRES were more frequent in the NOPHO cohort compared to those of the countries using BFM-protocols (OR = 2.14, CR95% (1.25–3.67), OR = 2.98, CI95% (1.33–6.65)) (see Table S4e). SLS did not associate with the studied SNPs.

#### 3.1.2. Survival Analyses on the Neurotoxicity Case-Control Cohorts

OS and EFS were studied on cohorts with adverse neurological symptoms and in association with SNPs. A higher risk for death was associated with AE in the studied unmatched Hungarian cohort (HR = 2.51, CI 95% (1.32–4.76)). Among the 82 AE cases, in our database two cases died related to neurotoxicity (9.5% of all exits). Examining SNPs with survival on the unmatched Hungarian cohorts of AE or ATE, patients with *CYP3A5* rs4646450 T allele had worse outcome (both OS and EFS). This risk was even higher in patients with TT genotype. *CYP3A4* rs3735451 GG genotype associated with poorer OS and EFS (see Tables S2b and S5a). Analyzing the Combined matched cohort of ATE in which only 5 SNPs were genotyped, *GSTP1* rs1695 GG + AG genotype was associated with better outcome (OS), and this association remained significant in the seizure sub-phenotype cohort, and in the ATE cohort during Induction-like cycles (see Tables S2b and S5b). Analyzing EFS of the Combined cohort in PRES, the worse outcome was associated with *ABCB1* rs2032582 TT genotype and with the combination of *ABCB1* rs1045642 TT genotype with *ABCG2* rs2231142 CA or AA genotypes (see Tables S2b and S5c).

### 3.2. Central Nervous System Relapse

We analyzed the impact of SNPs in metabolizing enzymes and transporters on the prevalence of CNS relapse, using the Combined relapse case-control cohort. When comparing patients with isolated or combined CNS relapse to non-relapsed controls, the *ABCB1* rs2032582 GT and the rs1128503 TT + CT genotype seemed to be protectors against CNS relapse. The results are shown in Tables S3a and S6a. Analyzing the survival of the Combined relapse case-control cohort, we have not found any significant SNPs in association with CNS relapse. The summary of the results is shown in Table S3b. The full set of results can be found in Table S6b.

### 3.3. Inverse Association of SNPs with Chemotherapy Related Adverse Neurological Events and CNS Relapse

**Figure 3:**
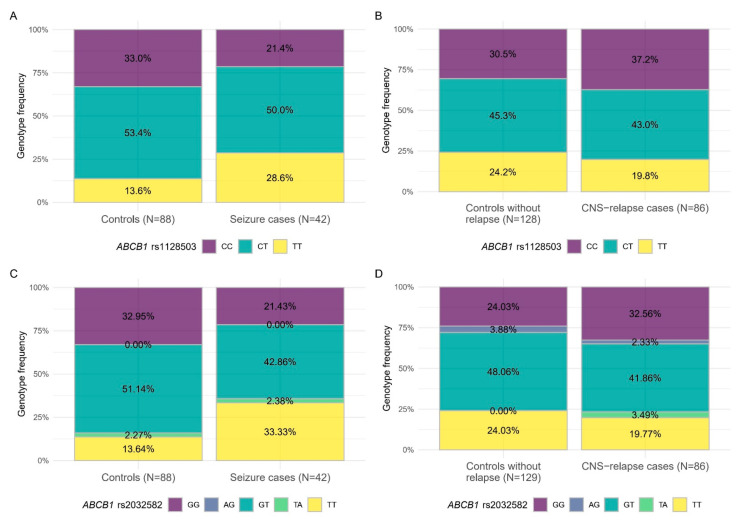
Inverse associations of blood-brain-barrier SNPs with toxic seizure or CNS relapse in case-control analyses. The studied populations were the Combined case-control cohorts of ATE and CNS relapse, respectively. (**A**) Genotype frequencies between cases and controls regarding association of *ABCB1* rs1128503 and seizure, (**B**) Genotype frequencies between cases and controls regarding association of *ABCB1* rs1128503 and CNS relapse, (**C**) Genotype frequencies between cases and controls regarding association of *ABCB1* rs2032582 (triallelic) and seizure, (**D**) Genotype frequencies between cases and controls regarding association of *ABCB1* rs2032582 (triallelic) and CNS relapse. Colors refer to genotypes.

Examining Combined cohorts of ATE and CNS relapse including case-control matched cohorts from all groups, we have found that patients with *ABCB1* rs1128503 TT or rs2032582 TT genotypes were more prone to have toxicity related seizures but lower incidence of CNS relapse. For more details see Figure 3 and Table 4.

**Figure 4:**
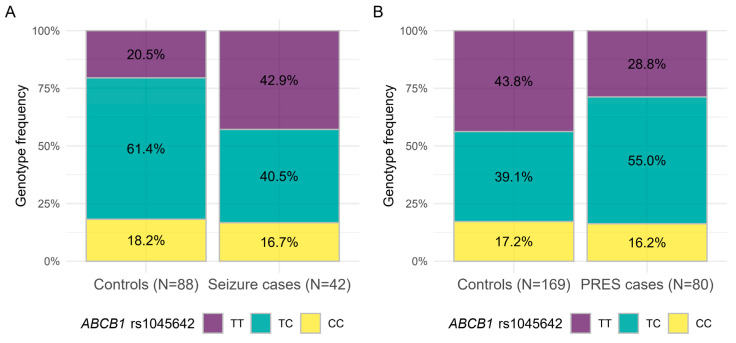
Genotype distributions of *ABCB1* rs1045642 in seizure or PRES Combined cohorts. (**A**) Genotype frequencies between cases and controls regarding association of *ABCB1* rs1045642 and seizure, (**B**) Genotype frequencies between cases and controls regarding association of *ABCB1* rs1045642 and PRES. Colors refer to genotypes.

*ABCB1* rs1045642 TT was also in inverse association with seizure and PRES in the Combined cohort (*p* = 0.011, OR = 0.34, CI95% (0.15–0.78), *p* = 0.017, OR = 2.10, CI95% (1.14–3.87), respectively) (Figure 4).

## 4. Discussion

In this study, we evaluated the association of SNPs in drug-metabolizing and transporting genes with acute CNS toxicity and CNS relapse episodes in patients with childhood acute lymphoblastic leukemia. In the Hungarian cohort, we found that *ABCB1* rs1045642, rs1128503, and rs2032582 TT genotypes, the combination of *ABCB1* rs1045642 TT genotype with *ABCG2* rs2231142 CA or AA genotypes, and *GSTP1* rs1695 AA genotype may increase the risk of chemotherapy-related adverse neurological symptoms. These associations were not confirmed in the Austrian-Czech-NOPHO Joined validation cohort, however, still appeared as significant in the seizure subgroup of the Combined cohort. Interestingly, there appears to be an inverse association of the SNP rs1045642 with PRES and seizure in Combined cohorts. Our results with *ABCB1* rs1128503 and rs2032582 in relation with seizure and CNS relapse suggest that blood-brain-barrier drug transporter gene-polymorphisms might have an inverse association with CNS toxicity and CNS relapse. The Hungarian AE cases had lower OS, *CYP3A5* rs4646450 and *CYP3A4* rs3735451 associated with worse OS and EFS in the Hungarian AE and ATE cohorts.

Patients with CNS toxicity had worse survival than control patients in our analysis. The direct contribution of neurotoxic events to the deaths were negligible. This is in parallel with findings of other studies and may be related to treatment delays, dose-reductions or omissions of intrathecal or systemic chemotherapy after the neurotoxic event, or enzyme inducing antiepileptic therapies increasing the metabolism of chemotherapy [53,54,55,56]. Delays in intrathecal MTX treatment caused by MTX neurotoxicity associated with increased risk of CNS relapse [31]. Similar strategies were applied indeed unfortunately in some hospitals at the time when our study cohort was treated [56].

ABC transporters are important in the resistance to methotrexate, cytarabine, vincristine, anthracyclines, and dexamethasone, influence response to treatment and survival [57,58,59]. Genetic variants of *ABCB1* were studied in hematological malignancies, a broad variety of conclusions regarding their function was observed but their true clinical impact is still under debate [60]. *ABCB1* rs1045642 TT + CT vs CC alleles were associated with higher 24 h plasma MTX concentration [61]. In contrast, rs1045642 CC genotype associated with higher MTX plasma level and with relapse investigating high risk ALL patients in another study [62]. *ABCB1* genetic variants can influence cerebrospinal fluid (CSF) drug levels. The CSF concentration of MTX was different between the rs1045642 C allele (CC + CT) carriers and TT homozygous patients [63]. *ABCB1* SNPs were found to associate with vincristine-related neurotoxicity in a study, however, they have found no association with SNPs included in our analysis [64]. Another *ABCB1* SNP, rs4728709 T allele was also protective against neurotoxicity in the study of Ceppi et al. [22]. *ABCB1* rs1045642 (C3435T) and rs2032582 (G2677T) TT genotype associated with worse EFS and the same trend was observed if rs1128503 T allele was also included in the analysis [22]. *GSTP1* protects against oxidative stress, *GSTP1* rs1695 is a missense variant, decreases the enzyme activity [65]. *GSTP1* rs1695 GG genotype associated with CNS toxicity and also with attention deficit in ALL survivors [19,66]. *GSTP1* rs1695 G allele in two different studies increased and reduced the risk for CNS relapse in ALL [15,67,68,69,70].

This study has multiple limitations. The retrospective data collection may have resulted in skewed populations. Another difficulty lies in the categorization of neurotoxic events into phenotype subgroups (like SLS, seizures, PRES), or into etiology groups (toxic or secondary). Various subsets of tests were missing (not performed or not available in retrospect) for some of the neurotoxicity cases, e.g., blood pressure, miscellaneous laboratory results and imaging. The differentiation of toxic PRES (direct drug toxicity) and secondary PRES (e.g., PRES evoked by hyponatremia or hypertension which had been caused by chemotherapy) is especially difficult and may just be theoretical. We aimed to be consistent and applied the logic described in Figure 1 and Supplementary Materials Patient Criteria and also took both the original opinion of the treating physician and the Delphi definitions [26] into consideration. The categorization of events was unambiguous in the large majority of the cases, so we think these factors don’t undermine the results of the study. Discrepancies in CNS2 status classification among study groups are well known, this confounding factor could not be avoided. Treatment heterogeneity may also have influenced our results. NOPHO protocols use higher and more frequent dosing of vincristine than BFM-based protocols applied in the other groups, the high rate of PRES among NOPHO patients may also relate to this.

Despite some identified associations that are concordant with our original hypothesis, we can’t formulate practical clinical guidance based on our results. As neurotoxic episodes proved to be reversible with only very few exceptions, surely CNS relapse should be the main focus in any future attempt on therapy individualization. There is great need for even larger international studies for studying these very rare events in more homogenous cohorts.

## 5. Conclusions

In the present study SNPs were investigated in a European international collaboration to assess their role in CNS complications and related survival. Key BBB genes *ABCB1* and *GSTP1* and their SNPs rs1045642, rs1128503, rs2032582 and rs1695 came to focus. Our findings suggest that genetic variations which are associated with a lower or higher risk of CNS complications can also associate with better or worse outcomes in respect of survival, respectively.

## Figures and Tables

**Table 1:** Basic characteristics of the studied populations with acute encephalopathy (AE) and acute toxic encephalopathy (ATE).

Study Cohort	Hungarian			Austrian	Czech	NOPHO ^4^	Combined
				Joined Validation Cohort			
	Non-matched		Matched	Matched			Matched
Phenotype	AE	ATE	ATE	ATE			ATE
Number of patients *n*	626	580	108	62	119	137	426
ATE Cases/controls *n* (%)	82/544	36/544	36/72	21/41	39/80	47/90	143/283
	(13/87)	(6/94)	(33/67)	(34/66)	(49/51)	(34/66)	(34/66)
Seizure only *n*	21	20	20	8	10	6	44
SLS ^1^ *n*	6	6	6	1	6	7	20
Toxic PRES ^2^ *n*	3	3	3	12	18	33	66
Gender *n* (%)	339	317	52	26	53	74	205
Male	(54)	(55)	(48)	(42)	(45)	(54)	(48)
Period of ALL diagnosis *y*	1990–2015	1990–2015	1992–2015	2010–2018	2003–2017	2008–2015	1992–2018
Age at diagnosis *n* (%)	104	88	35	30	42	29	136
>10 *yr n*	(17)	(15)	(32)	(48)	(35)	(21)	(32)
Median (range) *yr*	5.0 (1–18)	5.0 (1–18)	7.7 (1–18)	9.9 (1.8–17.7)	7.1 (1.3–18)	7.0 (1–16)	7.6 (1–18)
Risk group (HR ^3^) *n*	75	69	17	29	15	41	102
(%)	(12)	(12)	(16)	(47)	(13)	(30)	(24)

Abbreviations: AE: acute encephalopathy; ATE: acute toxic encephalopathy; ^1^ SLS: Stroke-like syndrome; ^2^ PRES: Posterior re-versible encephalopathy syndrome; ^3^ HR: high risk, as per patient’s treatment protocol; ^4^ NOPHO: Nordic Society for Pediatric Hematology and Oncology.

**Table 2:** Basic characteristics of the studied population of posterior reversible encephalopathy syndrome (PRES).

Study Cohort	Austrian	Czech	Hungarian	NOPHO ^2^	Combined
Matched cohorts					
Number of patients *n*	39	62	18	132	251
Cases/controls *n* (%)	13/26	19/43	6/12	44/88	82/169
	(33/67)	(31/69)	(33/66)	(33/66)	(33/67)
Gender *n* (%)	18	43	9	76	146
Male	(46)	(69)	(50)	(58)	(58)
Period of ALL diagnosis *y*	2010–2017	2003–2017	1998–2013	2008–2015	1998–2017
Age at diagnosis *n* (%)	14	16	9	23	62
>10 yr *n*	(36)	(26)	(50)	(17)	(25)
Median (range) *yr*	9.0(1.8–16.9)	5.68(1.3–14.5)	10.5(4–15)	8.0(1–15)	8.0(1–16.9)
Risk group (HR ^1^) *n*	21	7	3	48	79
(%)	(54)	(11)	(17)	(36)	(32)

Abbreviations: ^1^ HR: high risk; ^2^ NOPHO: Nordic Society for Pediatric Hematology and Oncology.

**Table 3:** Basic characteristics of the studied population of central nervous system first relapse (CNS relapse).

Study Cohorts	Austrian	Czech	Hungarian	NOPHO ^4^	Combined
Matched cohorts					
Number of patients *n*	8	152	60	100	320
Isolated CNS ^1^ relapse	1	10	4	19	35
Combined CNS relapse	2	26	12	12	51
Isolated BM ^2^ relapse	5	54	16	30	105
Relapse- free controls	0	62	28	39	129
Gender *n* (%)	4	102	42	62	210
Male	(50)	(67)	(70)	(62)	(66)
Period of ALL diagnosis *y*	2010–2014	1996–2017	1992–2013	2008–2015	1992–2017
Age at diagnosis *n* (%)	3	29	22	24	78
>10 yr *n*	(40)	(19)	(37)	(24)	(24)
Median (range) *yr*	9.5 (5.8–15.9)	4.2 (0.1–17.8)	7.4 (1–17)	5.0 (1–16)	4.9 (0.1–17.8)
Risk group (HR ^3^) *n*	5	38	17	27	87
(%)	(63)	(25)	(28)	(27)	(27)

Abbreviations: ^1^ CNS: central nervous system; ^2^ BM: bone marrow; ^3^ HR: high risk ^4^ NOPHO: Nordic Society for Pediatric Hematology and Oncology.

**Table 4:** Summary of the results of toxic seizure and CNS relapse analyses in Combined cohort.

Study Cohorts			Seizure	CNS Relapse Cases vs. Patients without Relapse (*n* = 86/129)
(Cases/Controls)			(*n* = 44/89)	
Gene	SNP	Comparisons	OR (CI95%)	OR (CI95%)
***ABCB1***	**rs1128503**	**TT + CT vs. CC**	2.10 (0.82–5.39)	***0.48 (0.24–0.96)***
		**TT vs. CC + CT**	2.49 (0.99–6.26)	0.74 (0.33–1.64)
		**CT vs. CC**	1.67 (0.61–4.52)	0.48 (0.23–1.01)
		**TT vs. CC**	***3.50 (1.10–11.12)***	0.46 (0.18–1.16)
	**rs2032582 (triallelic)**	**AG vs. GG**	nv	0.54 (0.10–2.97)
		**TA vs. GG**	2.16 (0.16–28.70)	nv
		**TT vs. GG**	***3.71 (1.23–11.17)***	0.59 (0.25–1.40)
		**GT vs. GG**	1.37 (0.50–3.75)	***0.41 (0.20–0.87)***

Abbreviations: nv: not valid; CNS: central nervous system; REL: relapse. Results with *p* ≤ 0.05 are shown with bold italics characters, significant results with *p* ≤ 1.13 × 10^−2^ are shown with bold characters.

## Data Availability

The datasets analyzed during the current study are available from the corresponding author on reasonable request.

## Supplementary Materials

The following are available online at https://www.mdpi.com/article/10.3390/cancers13102333/s1, Supplementary Patient Criteria, Table S1a: Information about the studied SNPs, Table S1b: Confounders and stratification listed for every analyses, Table S1c: Primer sequences used in Sanger-sequencing of the Austrian population, Table S2a: Results of the case-control analysis of chemotherapy related adverse neurological events, Table S2b: Survival analysis on the CNS adverse event case-control cohort, Table S3a: SNPs analyzed in the CNS relapse Combined case-control cohort, Table S3b: Results of the survival analysis of CNS relapse Combined cohort, Table S4a: Results of acute encephalopathy and acute toxic encephalopathy case-control study in the Hungarian cohort, Table S4b: Results of acute toxic encephalopathy case-control study in the joined validation cohort, Table S4c: Results of acute toxic encephalopathy case-control study in the combined cohort, Table S4d: Results of PRES case-control study in the combined cohort, Table S4e. Results of treatment protocol-comparisons among acute toxic encephalopathy subphenotypes in the combined cohort, Table S5a: Results of the survival study in the Hungarian cohort with acute encephalopathy and acute toxic encephalopathy, Table S5b: Results of the survival study in the combined cohort with acute toxic encephalopathy, Table S5c: Results of the survival study in the combined cohort with PRES, Table S6a: Results of case-control study in combined cohort with CNS relapse, Table S6b: Results of survival study in combined cohort with CNS relapse, Table S7: Applied protocols in the studied populations.
