# Supplementary material for: Pharmacogenetics of the Central Nervous System—Toxicity and Relapse Affecting the CNS in Pediatric Acute Lymphoblastic Leukemia
Source: Cancers (Basel). 2021 May 12;13(10):2333. doi: 10.3390/cancers13102333 (PMC8151239; doi:10.3390/cancers13102333)
Supplement: Supplementary file 1 [file cancers-13-02333-s001.zip › Supplementary_Patient_Criteria.pdf]

PATIENT CRITERIA

ACUTE TOXIC ENCEPHALOPATHY PROJECT

SELECTION OF CASES

Inclusion criteria

- Patients treated with frontline paediatric acute lymphoblastic leukaemia protocols
- Age 1-18 at diagnosis
- Patients with seizures, stroke like syndrome, PRES, depressed level of consciousness and those with overlapping CNS toxicity features of these above, but only those with CNS symptoms CTC grade ≥3, suspected to be evoked directly by neurotoxic chemotherapy, occurring in the time period between the first i.v. or i.th. chemotherapy dose and maximum 3 weeks after after the very last i.v. chemotherapy.

Exclusion criteria

- No available DNA
- Patients with comorbidities, medical history or co-medication that may influence the occurrence of CNS complications or their symptoms or trouble that diagnosis
- Patients with comorbidities, medical history or co-medication that may influence drug pharmacokinetics (e.g. chronic renal or liver disease, chemotherapy before the diagnosis of ALL, etc)
- Identified possible non-chemotherapeutic cause of the CNS symptoms/complications
  * CNS ALL-involvement (at diagnosis of ALL)
  * cerebrovascular events
  * CNS infections
  * serum Na<126 mmol/L, corrected Ca <1.9, other relevant electrolyte disturbances
  * hypoglycaemia (glucose <2,5 or >30 mmol/L) or hyperammonaemia or other metabolic causes
  * severe hypertension: systolic blood pressure 25 mmHg above the 90th centile at repeated measurements (at least 2)
  * possible CNS hypoperfusion or hypoxia

SELECTION OF CONTROLS

2 controls without CNS toxicity for each case:

Inclusion criteria

- Patients treated with frontline paediatric acute lymphoblastic leukaemia protocols
- Full treatment protocol completed
- Age 1-18 at diagnosis

Exclusion criteria

- CNS toxicity of any grade during ALL-treatment, even uncertain toxicities
- No available DNA
- Relevant deviation from treatment protocol
- Patients with comorbidities, medical history or co-medication that may influence the occurrence of CNS complications or drug pharmacokinetics.
- Previous chemotherapy before the diagnosis of ALL

HIERARCHY OF POINTS WHEN SELECTING CONTROLS
1. Treatment protocol and arm to match
2. Age +/-2 years if possible
3. Sex
4. Date of diagnosis

PRES PROJECT

SELECTION OF CASES
<u>Inclusion criteria</u>
- Patients treated with frontline paediatric acute lymphoblastic leukaemia protocols
- Age 1-18 at diagnosis
- Patients with PRES with CNS symptoms CTC grade ≥3, occurring in the time period between the first i.v. or i.th. chemotherapy dose and maximum 3 weeks after after the very last i.v. or i.th. kemotherapy.

<u>Exclusion criteria</u>
- No available DNA
- Patients with comorbidities, medical history or co-medication that may influence the occurrence of CNS complications or their symptoms or trouble that diagnosis
- Patients with comorbidities, medical history or co-medication that may influence drug pharmacokinetics

SELECTION OF CONTROLS
2 controls without CNS toxicity for each case:
<u>Inclusion criteria</u>
- Patients treated with frontline paediatric acute lymphoblastic leukaemia protocols
- Full treatment protocol completed
- Age 1-18 at diagnosis

<u>Exclusion criteria</u>
- CNS toxicity of any grade during ALL-treatment, even uncertain toxicities
- No available DNA
- Relevant deviation from treatment protocol
- Patients with comorbidities, medical history or co-medication that may influence the occurrence of CNS complications or drug pharmacokinetics.
- Previous chemotherapy before the diagnosis of ALL

HIERARCHY OF POINTS WHEN SELECTING CONTROLS
1. Treatment protocol and arm to match
2. Age +/-2 years if possible
3. Sex
4. Date of diagnosis

CNS RELAPSE PROJECT

SELECTION OF CASES
<u>Inclusion criteria</u>
- Patients with isolated or combined CNS relapses (1st relapse only) of ALL

- Patients previously treated with standard frontline paediatric acute lymphoblastic leukaemia protocols
- Age 0-18 at diagnosis
- No major deviation from the frontline ALL treatment protocol

Exclusion criteria

- No available DNA
- Patients with comorbidities, medical history or co-medication that may influence drug pharmacokinetics
- Previous chemotherapy before the diagnosis of ALL

SELECTION OF CONTROLS

2 non-relapsed and 1 isolated bone marrow relapse control for each case:

Inclusion criteria

- Patients treated for ALL with standard frontline paediatric protocols
- Age 0-18 at diagnosis
- No major deviation from the frontline ALL treatment protocol
- No CNS-relapse on ≥ 5 year follow up

Exclusion criteria

- No available DNA
- Patients with comorbidities, medical history or co-medication that may influence drug pharmacokinetics
- Previous chemotherapy before the diagnosis of ALL

HIERARCHY OF POINTS WHEN SELECTING CONTROLS

1. Treatment protocol and arm to match
2. ALL-immunophenotype
3. Age +/-2 years at 1st diagnosis of ALL if possible
4. Sex
5. Relevant leukaemia biology to match
6. Date of diagnosis
